# Exposure to *Aspergillus* in Home and Healthcare Facilities’ Water Environments: Focus on Biofilms

**DOI:** 10.3390/microorganisms7010007

**Published:** 2019-01-05

**Authors:** Malcolm Richardson, Riina Rautemaa-Richardson

**Affiliations:** Mycology Reference Centre Manchester, Wythenshawe Hospital, Manchester University NHS Foundation Trust, Manchester M23 9LT, UK; riina.richardson@manchester.ac.uk

**Keywords:** *Aspergillus* species, *Aspergillus fumigatus*, drinking water, biofilms

## Abstract

*Aspergillus* conida are ubiquitous in the environment, including freshwater, water for bathing, and in drinking water. Vulnerable patients and those suffering from allergic diseases are susceptible to aspergillosis. Avoidance of *Aspergillus* is of paramount importance. Potential outbreaks of aspergillosis in hospital facilities have been described where the water supply has been implicated. Little is known regarding the risk of exposure to *Aspergillus* in water. How does *Aspergillus* survive in water? This review explores the biofilm state of *Aspergillus* growth based on recent literature and suggests that biofilms are responsible for the persistence of *Aspergillus* in domestic and healthcare facilities’ water supplies.

## 1. Introduction

Aspergillosis is caused by the filamentous mould *Aspergillus*. The term describes a wide spectrum of diseases from invasive disease to evoking allergic responses [1,2]. The disease can occur in most body organs in humans and animals. Humans have highly efficient innate and immune mechanisms to prevent themselves from being infected by *Aspergillus* species. It is when these mechanisms are defective or absent that *Aspergillus* can grow within the body. Most people who may develop life-threatening aspergillosis are those with a weakened or compromised immune system due to corticosteroid therapy, due to being managed in intensive care, following transplant surgery, or as a consequence of cancer therapy. In contrast, aspergillosis can occur in immunocompetent individuals with no known suppression of their immune system, where previous tissue damage has occurred due to heavily scarred pulmonary tissue as a result of tuberculosis, chronic obstructive pulmonary disease, bronchiectasis where the airways become abnormally widened, or some other underlying pulmonary disease.

Many different manifestations of aspergillosis are well-described, including allergic aspergillosis (rhinosinusitis and bronchopulmonary) (>10 million worldwide), chronic pulmonary aspergillosis (~3 million worldwide), invasive aspergillosis (incidence >300,000 annually), and superficial disease (notably keratitis, otomycosis, and trauma or burn wound infections) [1,2]. These conditions are seen worldwide (https://www.gaffi.org/).

The genus *Aspergillus* includes over 300 species. *Aspergillus* spores (conidia) are commonly found in outside air, water, food items, soil, plant debris, rotten vegetation, manure, sawdust litter, bagasse litter, animal feed, bark chippings, on animals, and in the built environment. Exposure to *Aspergillus* occurs worldwide, mainly by inhalation from the outdoor or built environment. Water may be an alternative source of *Aspergillus* conidia. This will be explored in this review.

Around 20 species have so far been reported as causative agents of opportunistic infections in humans, although many cryptic species are now implicated, so the term complex is added to species names, unless definitive identification has been conducted. Among these, *Aspergillus fumigatus* is the most commonly isolated species, followed by *A. flavus* and *A. niger. A. nidulans, A. terreus*, *A. calidoustus*, and *A. versicolor* are among the other species less commonly isolated.

Multi-locus DNA sequence analysis has enabled the description of previously unknown ‘cryptic’ *Aspergillus* species, whereas phenotypic-based identification of *Aspergillus* can only identify isolates down to the species complex [3]. There are two main features of these ‘cryptic’ *Aspergillus* species. Firstly, the prevalence of these in clinical samples is relatively high compared with other filamentous fungal taxa, such as the agents of mucormycosis, *Scedosporium*, or *Fusarium*. Secondly, it is mandatory to identify these species because of the high frequency of antifungal drug-resistance. Matrix-assisted laser desorption/ionization-time of flight mass spectrometry (MALDI-TOF MS) enables the identification of molds and yeasts with an accuracy similar to that of DNA sequence-based methods [4]. MALDI-TOF MS is well-suited to the routine clinical laboratory workflow and facilitates the identification of common and ‘cryptic’ *Aspergillus* species. However, more reliable databases have to be developed to accommodate these. The fungal databases are the key components of commercial MALDI-TOF platforms. Commercial databases contain a large collection of common fungi, but more unusual and ‘cryptic’ species are not well-represented. Open-source databases of unusual fungi are becoming more accessible. Moreover, these databases have to contain fingerprints of multiple strains of the same fungus. Further development of these and other platforms will lead to a better understanding of the epidemiology and clinical importance of these previously unrecognized *Aspergillus* species.

The mycobiome of indoor and external environments, for example, air, dust, and soil, has been studied in some detail and comparisons have been made with the mycobiome of the respiratory tract [5]. *Aspergillus* species have been found in varying proportions in both, but no one study has focussed on the link between the mycobiome of water and human disease.

## 2. Water as a Reservoir for *Aspergillus*

Water entering a building is not sterile. Homes and healthcare facilities typically receive potable water from their local public water system or municipality. Potable water is used extensively in the healthcare environment. It is used for drinking, patient bathing and showering, handwashing, rinsing medical devices, hydrotherapy pools, and to make ice. Potable water is not sterile. It contains a high bacterial load. Fungi also have frequently been isolated from drinking water [6]. Opportunistic pathogens represent a significant proportion of the microbiome of drinking water and have emerged as a significant public health issue. Drinking water also contains fungi pathogens, such as *Aspergillus* spp. and *Fusarium* spp. [6]. However, little is known about the dynamics of fungal communities during the treatment of drinking water. Studying fungal populations in drinking water over time should identify areas and times where *Aspergillus* may proliferate. There are large gaps in our knowledge regarding the dynamics of fungal ecology during the drinking water treatment process and final delivery, and operational factors that shape the community structure. Contaminated water presents a number of exposure pathways. Water-related fungal infectious risks exist in both healthcare and community environments. Individuals can be directly exposed to these organisms either through bioaerosols and water, after ingestion, inhalation, and skin contact, and through mucous membranes.

In 2016, the present authors highlighted the scarcity of studies on the topic of *Aspergillus* in drinking water and that there were a number of aspects that remained poorly understood [7]. There was a plea for research to further explore the importance of drinking water as an environmental source of fungi in vulnerable or at-risk population groups. The expectation was that greater knowledge on the importance of the ingestion of *Aspergillus* in drinking water, as opposed to inhalation, as an exposure pathway, will ensure that mitigation measures for at-risk patients are appropriate.

In 2017, an in-depth, exhaustive review on fungal contaminants in drinking water was published [6]. Here, we summarize the body of knowledge reviewed by these authors, and others, in relation to *Aspergillus* spp., followed by a focus on biofilms in domestic and healthcare facility water supplies.

Babić and colleagues reviewed how the chemical properties of water influence the growth and survival of fungi in water systems, and vice-versa [6]. Fungi are actively involved in the dissolution and corrosion of rocks and the precipitation of minerals. In general, rocks with an alkaline pH are more susceptible to fungal colonization than rocks with an acidic pH. In addition to limestone, a range of other rock types positively influence the growth of *A. niger*. Surface water is rich in the products of organic matter degradation, which promotes the growth of plant degrading filamentous fungi, including *Aspergillus* spp. In comparison, groundwater contains more inorganic ions and less organic matter and therefore fungi have not been isolated so frequently. Environmental water in areas that are densely populated contain high amounts of organic waste, as well as a number of pollutants, including organo-halogens, pesticides, and long-chain aromatic hydrocarbons such as benzene and xylene. *Aspergillus* species are able to break-down long-chained pollutants and have been isolated from contaminated waters. *Aspergillus* has also been isolated from waters heavily contaminated with bacteria and algae, which results in low oxygen concentrations. *Aspergillus* can grow under hypoxic conditions.

The effect of sunlight and water temperature on fungi is not well-documented. Babić and colleagues have reviewed the few studies that have been conducted [6]. The effect of solar-UV-radiation varies with the time of day, and is lower during cloudy days, in large volumes of water, and in water with high amounts of organic matter with increased turbidity. UV-radiation will also raise the water temperature. High levels of *Aspergillus* have been found in surface water during summer months, being replaced by other filamentous fungi during the cooler seasons.

Drinking water quality deteriorates during transportation through drinking water distribution systems (DWDS), reviewed in reference [8]. Microbial activity and ecology, particularly within biofilms that occur on the inner-pipe surface of DWDS, are emerging as important drivers in the degradation process. More recent reviews, for example, by Douterelo and colleagues [9,10], reinforce the view that the delivery of high-quality, potable drinking water primarily depends on the optimal operation of the DWDS. DWDS are complex pipe networks which function as discrete ecosystems which are dominated by microorganisms that are attached to the inner pipe surfaces and grow into the lumen of the pipes. Microbial dynamics in any ecosystem are determined by interactions between microorganisms and the surrounding environment. However, within these water transportation systems, the dynamics of microbial populations and a change in their composition over time remain largely unexplored.

Water after filtration is usually still not suitable for human drinking. Additional primary and secondary disinfection, such as UV-irradiation and ozonation, is often used. UV-irradiation does not appear to be optimal for *A. fumigatus*. Ozone does appear to be effective against a range of fungi, including *A. fumigatus*. Both processes are usually combined with chlorination. However, *A. fumigatus* appears to be resistant to calcium hypochlorite in some studies [6].

Despite well-developed raw water cleaning processes, fungi have been discovered in tap water systems as single cells or hyphal fragments, and as a part of biofilms, reviewed in reference [11]. An accumulation of research studies from 19 European countries has shown the difference between fungi communities in surface water and groundwater versus tap drinking water [11]. More than 400 different species have been found to inhabit different water sources: *Aspergillus* species were reported from 17 out of 19 countries (89.5%), followed by *Cladosporium* and *Penicillium* (both were reported from 84.2% of countries), *Trichoderma* (73.7%), *Alternaria* and *Fusarium* (both 68.4%), and *Aureobasidium* and *Mucor* (both 52.6%). Most fungi were isolated from both raw water sources (surface- and groundwater) and tap water, while species from the genera *Mucor*, *Trichoderma*, and *Penicillium* were found more often in surface water samples. These studies were carried out mainly using traditional cultivation techniques and may thus not be inclusive. Next generation sequencing techniques have the potential to elucidate the mycobiome of water supplies even further.

When employing direct microscopy, fungal elements can be seen in biofilms; however, it is virtually impossible to definitively identify the species unless the mould is sporulating. Next-generation sequencing of biofilms has identified opportunistic yeasts such as *Candida albicans, C. parapsilosis,* and *Exophiala dermatitidis*, but has scarcely mentioned *Aspergillus* species [11,12]. An understanding of fungal biofilms in water supplies has been the main focus of water mycology over the past two years [11,12]. Because of the expected climate change, it is probable that in the future, the earth’s warming will lead to a rise in the temperature of surface water, water distribution systems, and pipe networks. Opportunistic fungi, including *Aspergillus* species, which are capable of growing in drinking water, are often adapted to higher temperatures. Several species of *Aspergillus* are thermotolerant. As temperatures rise, these organisms could therefore occur more frequently in the microbial populations in drinking-water-related environments.

### 2.1. Diversity of Fungi in Water—Regional

As an illustrative example, the load and presence of environmental, mycotoxin-producing, and potentially pathogenic fungi in man-made water systems (domestic dwellings, hospitals, and shopping centers) connected to the municipal water distribution network in Istanbul, Turkey, was investigated [13]. The mean fungal load found in different water samples was 98 colony-forming units (CFU)/100 mL of water in shopping centres, 51 CFU/100 mL in hospitals, and 23 CFU/100 mL in homes. The dominant fungal species were *Aureobasidium pullulans* and *Fusarium oxysporum*. Aflatoxigenic *A. flavus* and ochratoxigenic *A. westerdijkiae* were only detected in hospital water samples. *Alternaria alternata, A. clavatus, A. fumigatus*, and *Cladosporium cladosporioides* were also detected in the samples. This study demonstrated that in a big metropolitan area, water supplies contained environmental, pathogenic, allergenic, and mycotoxin producing fungi. It was concluded that the current disinfection policies and procedures in place were inadequate.

A number of studies from different regions of Europe have underscored the great biodiversity of fungi surface sources and groundwater, reviewed in [12]. Several pathogenic species have been found, including *A. fumigatus*, and rarer species known to be less sensitive to antifungal agents, including *A. calidoustus* and *A. viridinutans*. The dominance of *Aspergillus* species in these and other studies could reflect their high frequency in the surrounding air, reviewed in [6].

### 2.2. Water Systems—Freshwater

Fungi are much less common in freshwater biomes predominated by bacteria (often present as biofilms), cyanobacteria, archaea, algae, viruses, and protozoa, reviewed in [14]. Nonetheless, microscopic fungi are present in freshwater communities, although they exist in relatively lower concentrations, are rarely planktonic (free-living), and are even more rarely pathogenic.

Water could be the preferred habitat of certain fungal species. A quantitative evaluation of the taxa isolated from water was shown to depend on its origin: surface water presents a higher risk of colonization compared to deeper samples, such as groundwater [15].

Potentially pathogenic fungal microorganisms are found in a variety of freshwater sources, including surface waters, drinking water, and public bathing and swimming facilities. Fortunately, fungal infections as a result of freshwater exposure or trauma are rare. The most common entities appear to be fungal keratitis, otitis externa, and tinea pedis. Well-documented reports describe deep fungal infections resulting from freshwater exposures following natural disasters or near-drowning episodes. As with most cryptic fungal infections, freshwater-related or otherwise, this etiology should be suspected when bacterial cultures or molecular tests are normal or when the infection inexplicably worsens or fails to resolve with appropriate antibacterial therapy.

Like *Legionella pneumophila* and other bacteria, fungi may be associated with or be harbored by free-living amoebas in freshwater, including tap and potable water [16]. The importance of this association is still being elucidated. In addition to adjacent soil, one source of freshwater fungi contamination by fungi is atmospheric dust (dispersed by wind, excavation, and other soil-disturbing activities) [5]. It is to be expected that there are regional variations in species diversity. A variety of potentially pathogenic fungi may be found in various bodies of water, sometimes as the result of runoff or sewage contamination. The presence of some fungal contaminants in drinking water may be related to their establishment of biofilms in portions of the water distribution system upstream from household or commercial establishment plumbing systems, as discussed below. Hageskal and colleagues provided an informative review of the methodological challenges and difficulty in interpretation of microbiological assessments [17].

## 3. Aspergillus Biofilms

There are a number of key factors to consider [11]: (1) Water entering a home or healthcare facility is not sterile; (2) the design of a domestic or hospitals plumbing system and patterns of water use allow biofilms to form; (3) fungal pathogens establish themselves as biofilms in plumbing networks; (4) fungal pathogens associated with plumbing biofilms have been epidemiologically linked to healthcare acquired infections; and (5) risk of infection can be reduced through the development and implementation of a water management program.

Recent studies have suggested that biofilm formation by *A. fumigatus* in humans may be one of the most important virulence factors in chronic pulmonary aspergillosis, invasive disease, and fungal balls in the sinuses and lungs (aspergilloma) [18]. Fungal biofilms forming on host surfaces or cells are comprised of whole cells, cell wall components, secondary metabolites, drug transporters, and extracellular material. The biofilm phenotype of the fungus is refractory to most conventional antifungals. Thus, over the past few years, an in-depth analysis and understanding of *A. fumigatus* biofilms has been carried out to devise newer and better antifungal targets for treating complex *A. fumigatus* biofilm-associated diseases. Does what we know about *Aspergillus* biofilms in the clinical setting translate to *Aspergillus* biofilms in water delivery systems in the home and healthcare facilities?

A number of laboratory studies may provide some pointers as to how *Aspergillus* biofilms grow in the lumen of water distribution systems [19]. The formation of *A. fumigatus* biofilms is similar, regardless of the provenance of the isolate, but differences are apparent according to the ambient temperature. Stages of biofilm development include the following: Firstly, adhesion to an inanimate surface (typically over a 4 h period) followed by cell co-aggregation and the formation of extracellular matrix substance (EMS); secondly, conidial germination into germlings (8–12 h—for example, *A. fumigatus* conidia will germinate within 6 h under favourable conditions), followed by hyphal development, hyphal elongation, and expansion with channel formation (16–20 h); thirdly, biofilm maturation as follows: mycelia development, stratifying of hyphal layers, channel formation within these hyphal matrixes, and high structural arrangement of the mycelia that include anastomosis of adjacent hyphae and extensive production of EMS (24 h). The EMS covers, surrounds, and strengthens the mycelial meshwork, particular at 37 °C. When using clinical isolates, irregular fungal structures, such as micro-hyphae that are short and slender hyphae seem to occur; finally, regarding cell dispersion, soil isolates exhibit higher conidial formation than clinical isolates, which have the capacity to germinate and generate new mycelia growth. Shedding of fungal elements from the biofilm most likely occurs. It is highly likely that *Aspergillus* attaches to pre-existing bacterial biofilms, for example, *Pseudomonas aeruginosa*, which is often considered as the architect of the biofilm, due to its ability to produce an exopolysaccharide or glycocalyx matrix. It is highly likely that other filamentous fungi form biofilms in a similar way, for example, *Fusarium*.

### 3.1. Optimal Conditions for the Formation of Aspergillus Biofilms

A report from the Netherlands has shown that the season of the year influences the growth of *Aspergillus fumigatus* [20]. Using molecular tools, it was demonstrated that fungi, including *A. fumigatus*, generally occur in drinking water in the Netherlands, and are capable of multiplying in distribution systems and indoor installations. Drinking water temperatures between 5 °C and 22 °C had no influence on the number of fungi. *A. fumigatus* appeared to be capable of multiplying in distribution systems and/or indoor installations. Drinking water temperatures around 20 °C resulted in higher counts of *A. fumigatus* than at temperatures around 7 °C. However, the authors emphasise that it is still unclear whether the occurrence of the strains of *A. fumigatus* found in water would cause clinical disease.

Prior to the time period of the current review, the concept of fungal biofilms in water supply systems was introduced [14]. The authors discussed the need for standardized methods to investigate water for fungi and presented data showing that fungi did form biofilms. The major part of the fungal biofilm biomass in drinking water distribution systems is attached to pipe surfaces. Several factors influence biofilm development, including temperature, nutrients, residual disinfectant, the hydraulic regime, and characteristics of the network surface/substratum. This work also demonstrated the concept of mixed fungal biofilms, a situation well-recognised with mixed fungi-bacteria biofilms in a number of clinical scenarios.

The materials used for manufacturing water supply networks have been shown to influence the microbiological quality of water and the formation of biofilms [6]. Network pipe systems are constructed from a range of materials which may interact with residual chlorine and chlorination by-products. These materials may also influence the microbiological quality of the water by promoting biofilm formation. In general, bacteria and fungi are more likely to form biofilms in pipe systems made from iron or steel, in comparison to PVC [6]. The lumen of the pipes can become rough, inducing changes in water flow and causing a reduction in shear forces, enabling the easy attachment of microbial cells. The number of fungal cells inside biofilms may be 5000 times higher than in running water. Under experimental conditions, *Aspergillus* biofilms were fully formed within 48 h from the start of an experiment mimicking real conditions in tap systems [6]. *Aspergillus* biofilms have been found in taps in private homes, hospitals, and industrial premises. Once established, fungal biofilms are difficult to eradicate from the pipe system, resulting in altered taste, odor, the production of allergenic compounds, and mycotoxins [11,12].

### 3.2. Implications of Aspergillus Biofilms in Drinking Water Distribution Systems

Fungi growing as biofilms inside taps and in drinking and cooking water will affect the taste and produce an odor which will directly impact the chlorination process in use, due to the release of a large number of products known as secondary metabolites (extrolites) [11]. These are very diverse and specific for different fungal species and some are known mycotoxins. It is clear that the role of secondary metabolites in specific ecological niches is to defend their habitat and suppress the growth of competitors. However, a number of mycotoxins may be detrimental to human health in higher concentrations or as a result of prolonged, chronic exposure. Fungal cell wall components, fungal allergens, and the fungal biomass itself may drive allergies and result in opportunistic and systemic infections, mainly in profoundly immunocompromised individuals [1]. Even though fungi are recognized as causative agents of systemic respiratory, mucosal, rhinocerebral, cutaneous, and subcutaneous infections, they remain largely overlooked in the regulations of water quality and human consumption. There are many possible reasons for this, including: a lack of knowledge of the concentration of fungi in water, different culture methods, and the low number of reports making connections between the presence of fungi in tap water and the occurrence of diseases in humans. Recent natural disasters, such as tsunamis and hurricanes, have highlighted the possibility of drinking water contamination with pathogenic fungi, including *Aspergillus*.

The identification of pathogenic fungi from water in healthcare facilities such as *Aspergillus, Fusarium, Penicillium, Scedosporium*, and agents of mucormycosis and the unavoidable formation of a polymicrobial biofilm in waterlines is of concern. To prevent a risk of fungal infection in this setting, health authorities have implemented a number of measures to mitigate this: ultraviolet radiation to treat the incoming water, continuous chemical treatment or thermal shock within the pipe network, or filtration at points of use, reviewed in [11]. It is highly likely that *Aspergillus* conidia are released from contaminated water in bioaerosols, or the aerosolization of a moldy aquatic niche (wet cell) constituting additional exposure pathways in addition to the familiar dispersal of conidia from visible and interstitial mould growth.

Following on from our understanding of how *Aspergillus* biofilms form and resist antifungal treatment in the human respiratory tract, an additional concern when discussing biofilms in water distribution systems is that within a biofilm, sessile microorganisms become less susceptible or sometimes totally resistant to different antimicrobial compounds, compared to microorganisms growing planktonically. This resistance is partially explained by the presence of persister cells, which are highly tolerant to biocides, antibiotics, and antifungals, especially in the deeper layers of the biofilms [11]. Persisters are dormant variants of regular conidia and hyphal structures that form stochastically in biofilms and are highly tolerant to antifungals. *Aspergillus* biofilms can be considered as reservoirs of fungal contamination of water systems by the periodic or sustained release of hyphal fragments or longer lengths of hyphae which are isolated or in clusters embedded in an extracellular matrix. Biofilms grow at an interface such as the one existing between air and water, and may sporulate. These conditions are found in water systems, and in community and hospital environments, for example, in shower heads. This is also the case in endoscopy reprocessing units, dialysis units, and dental units.

## 4. The Medical Implications of Exposure to *Aspergillus* in Water in Indoor Environments

**Figure 1:**
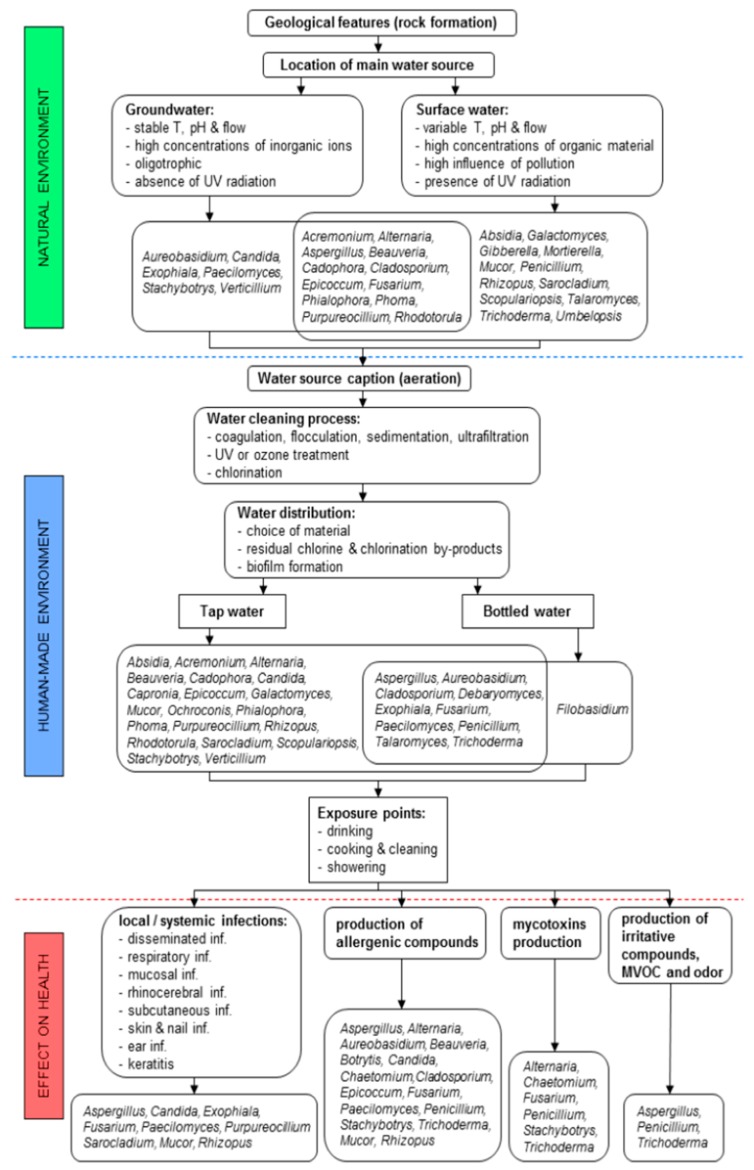
Abiotic, biotic, and anthropogenic factors influencing fungal presence in groundwater, surface water, tap water, and non-mineral bottled water, with a possible effect of fungi on human health via different exposure points. The most common factors having an influence on the fungal presence and diversity in different water sources are divided into factors influencing fungal presence, mainly in raw water sources in the natural environment (indicated with green colour), anthropogenic factors influencing fungal presence during the production of tap and non-mineral bottled water, and exposure points of fungi via water-related activities (indicated with blue colour). Red colour indicates the most frequently detected fungal genera from tap and bottled water with their possible effects on human health. Reproduced from [6], open access and with authors’ permission.

It is clear that water constitutes an exposure pathway. Individuals are exposed to pathogenic fungi, including *Aspergillus*, in a number of different situations: drinking water; bathing and showering; or indirectly due to the use of household appliances connected to the water supply, such as dishwashers and washing machines (Figure 1). Increasingly, a number of other environments are being recognised as potential sources of *Aspergillus*, such as bottled water, dental lines, and haemodialysis units (reviewed below).

### 4.1. Direct Contact with Aspergillus

Many fungi survive in moist environments. *Aspergillus* conidia can survive for many years when stored in water in the dark (unpublished data, M Richardson). *Aspergillus* biofilms can develop in shower hoses and shower heads. An association has been claimed between showering and the effect of bioaerosols containing *Aspergillus*, and exacerbation of asthma and hypersensitivity pneumonitis (extrinsic allergic alveolitis) [6]. Hospital water may also act as a source of hospital outbreaks of aspergillosis.

### 4.2. Indirect Contact with Aspergillus

Dishwashers and washing machines harbor a variety of filamentous fungi and black yeasts, but *Aspergillus* does not appear to feature in the mycobiome surveys of these appliances [11].

## 5. Potential Exposure to *Aspergillus* in Special Aqueous Environments

### 5.1. Swimming Pool Facilities

Contamination of swimming pools with pathogenic organisms and impact on human health has long been of concern. The possible transmission pathways of fungi in indoor swimming pool facilities have been assessed in different areas of swimming pool facilities by the culture and typing of the fungal isolates [21]. Air, water, and surface samples were collected from seven different indoor swimming pools. Species identification was based on DNA internal transcribed spacer (ITS) sequences. The maximum concentrations were found on surfaces, in water and air. Over 450 isolates were recovered, belonging to 111 fungal species, of which 50 were clinically relevant. *Phialophora oxyspora* (13.3%) and *Trichosporon dohaense* (5.0%) were the most frequently isolated species and mainly detected on floors, as were *Trichophyton interdigitale* and *T. rubrum*. *Penicillium* spp. and *Aspergillus* spp. were the dominant molds in water and air. The highest fungal concentrations posing the highest risk of contamination were areas where swimming pool visitors converged while moving from one room (e.g., dressing room) to another (e.g., shower room) and walking barefoot. As suggested in numerous other studies, the dispersal of fungi on floors is most likely facilitated by the pool visitors and cleaning machines of various types. The authors advocate that preventive measures such as cleaning should minimize the burden of clinically relevant fungi in swimming pools and similar ‘sub-tropical paradises’ since these potentially pose a health risk to vulnerable individuals, especially children and the elderly.

### 5.2. Haemodialysis Units

Haemodialysis is a type of treatment for kidney failure. It uses a haemodialysis machine, dialyzer, dialysis solution, catheters, and needles, all of which favor biofilm formation. Do pathogenic fungi form biofilms in dialysis equipment? To explore this, *Aspergillus* and *Fusarium* biofilms were grown in liquid culture containing dialysate or dialysate supplemented with glucose [22]. The biofilms were incubated at 30 °C for 72 h, quantified using violet crystal staining, and viewed under the transmission electron microscope. All the fungi formed biofilms under all test conditions. However, Bonferroni analysis (an adjustment made to *P* values when several dependent or independent statistical tests are being performed simultaneously on a single data set) revealed that the dialysate supported the growth of *Aspergillus*, whereas both the dialysate and dialysate supplemented with glucose promoted the development of *Fusarium oxysporum* biofilms. Scanning electron microscopy of biofilms that grew on catheters after 72 h revealed that *Aspergillus* had germinated and formed abundant hyphae; when *Aspergillus* was grown in the dialysate, an extracellular matrix was visible on the surface of some hyphae. The authors conclude that their study may contribute to the formulation of new strategies to monitor biofilm formation and to increase knowledge associated with fungal biofilms in the dialysis environment.

### 5.3. Showers

During showering, people are exposed to fungal propagules (conidia and hyphal fragments) via bioaerosols released into the environment [11,23]. Inhalation of water droplets containing these fungal components is the most relevant route of pulmonary and systemic infection for vulnerable patients. Any situation that enhances the air-borne dispersion of mould propagules increases the exposure of patients to such pathogens. Thus, special attention is paid to bioaerosols released in bathrooms in hospital environments. Recent research conducted on shower hose biofilms revealed the presence of a number of opportunistic pathogens: *A. glaucus*, *Cladosporium* spp., *Exophiala mesophila*, *Fusarium fujikuroi* species complex, *Malassezia restricta*, *Penicillium* spp., and *Schizophyllum commune* [6,23]. In a seminal study, Anaissie and colleagues reported a shift in the fungal population in the air and on surfaces between and immediately after showering [5]. Showering increased the presence of filamentous fungi, including *Aspergillus*. Molds were recovered in 70% of 398 water samples. The authors suggested that hospital water distribution systems may serve as a potential indoor reservoir of *Aspergillus* and other molds, leading to the aerosolization of fungal spores and potential exposure for patients.

### 5.4. Centralised Water Treatment Systems

Centralized water treatment plants are facilities where large volumes of water are treated at high flow rates in a “central” location and the water is then distributed via networks of pipelines, channels, and intermediate reservoirs. Centralized water treatment mainly operates in major urban areas of most parts of the developed world. A number of studies suggest that centralized drinking water treatment dictates the composition of the final drinking water microbial population via the selection of community members and that the eukaryotic community is controlled by physical treatment processes [24]. The effect of centralized water treatment processes on the diversity of fungal populations in drinking water has not been previously evaluated. Interestingly, it has been shown that the relative abundance of *Aspergillus* spp. significantly increased through the water treatment process, especially following disinfection, suggesting that this fungus is less efficiently removed by the conventional water treatment process or is more resistant to the selection pressure posed by water treatment processes. The relative abundance of *Aspergillus* spp. increased significantly from raw water to post-disinfection water. Interestingly, there was an increase in the relative abundance in water samples from post-filtration to post-disinfection. ‘Linear discriminant analysis Effect Size’ analysis also showed that *Aspergillus* spp. were significantly enriched post-disinfection.

### 5.5. Bottled Water

Ribeiro and colleagues conducted a 12-month survey of a drinking water bottling plant in Portugal to evaluate the diversity of the mycobiota [25]. The predominant fungal genera ranked in order of the highest numbers isolated were *Penicillium, Cladosporium*, and *Trichoderma*, followed by *Aspergillus, Paecilomyces*, and others. As expected, the highest numbers of isolates were collected during the warmer, late spring, and early summer months of the year. The authors advocated that during those times of the year when fungal contamination is high, filters should be changed on a regular basis. In order to assess whether contamination was from a focal or multiple points in the bottling facility, molecular methods were used specifically to identify *Penicillium brevicompactum*. Fungal contamination arose from multiple sources. Some *P. brevicompactum* strains were very similar in profile and were detected at different sampling times, indicating that they were endogenous to the bottling plant. There was no evidence to suggest that fungi detected in the source water contaminated other parts of the bottling plant. However, there was evidence that *P.* brevicompactum strains isolated from water filters were detected elsewhere in the plant, underscoring the importance of changing filters on a more regular basis during periods of high fungal contamination.

## 6. Conclusions

The recognition of *Aspergillus* biofilms in water delivery systems over many years has more recently helped to understand the formation of these fungal communities on and in various body spaces, leading to aspergillosis and presenting a formidable target for antifungal therapy. *Aspergillus* has been found to contaminate water supplies throughout Europe and beyond. It is not surprising that this filamentous fungus has the propensity to form communities on abiotic surfaces, such as water pipes. Furthermore, fungal biofilms have been seen to increase in complexity over time in water supplies, making them more difficult to eradicate.
